# Evolution of Chatbots as an Educational and Supportive Digital Intervention for Family Caregivers of People With Dementia: Protocol for a Systematic Integrative Review

**DOI:** 10.2196/91741

**Published:** 2026-06-09

**Authors:** Sarath Rathnayake, Ana Barbosa, Jiada Tu, Rebecca Randell

**Affiliations:** 1 Centre for Digital Innovations in Health and Social Care University of Bradford Bradford, West Yorkshire United Kingdom

**Keywords:** chatbot, conversational agent, dementia, education, family caregivers, support, integrative review, artificial intelligence, AI

## Abstract

**Background:**

Most people with dementia reside in the community and are cared for by family members. Family caregivers play an essential role in supporting their loved ones with dementia and require adequate education and support to address their care needs. In recent years, there has been growing interest in the use of chatbot technologies in health care, particularly to provide education and support for caregivers. However, evidence on the development, use, and effectiveness of these technologies in dementia care remains limited.

**Objective:**

This systematic integrative review aims to synthesize evidence on chatbot applications as an educational and supportive tool for family caregivers of people with dementia.

**Methods:**

A systematic integrative literature review will be conducted following a validated framework. The findings will be reported in accordance with PRISMA (Preferred Reporting Items for Systematic Reviews and Meta-Analyses) guidelines. The search strategy will be structured around 3 broad concepts: “dementia,” “caregiver,” and “chatbot applications.” Full-text articles based on empirical studies published in English from inception to 2026 that address the design, development, or evaluation of chatbot applications for family caregivers of people with dementia will be included. Editorials, study protocols, review articles, opinion and perspective papers, technical notes, and non-English publications will be excluded. Covidence software will be used to manage study screening. At least 2 independent reviewers will screen titles, abstracts, and full texts and extract data using a pretested data extraction form. Methodological quality will be assessed using Mixed Methods Appraisal Tool (version 2018), and data relevance will be evaluated using a 2-point scale (high or low).

**Results:**

The literature search will commence in May 2026, and the findings are expected to be published as a systematic integrative review, with submission planned for January 2027.

**Conclusions:**

This systematic integrative review aims to synthesize evidence from qualitative, quantitative, and mixed methods studies to provide a comprehensive understanding of how chatbot interventions are conceptualized, developed, and evaluated within dementia caregiving contexts. In addition, this review will identify key barriers and challenges as well as ethical and safety concerns associated with the design, development, and implementation of chatbot applications for family caregivers of people with dementia.

**Trial Registration:**

PROSPERO CRD420261288076; https://www.crd.york.ac.uk/PROSPERO/view/CRD420261288076

**International Registered Report Identifier (IRRID):**

PRR1-10.2196/91741

## Introduction

### Background

Dementia is a global health problem and an umbrella term encompassing a range of diseases characterized by a progressive decline in memory, cognitive abilities, and behavior, which interferes with an individual’s independence in activities of daily living [1]. Globally, dementia affected approximately 57 million people in 2021, with >60% living in low- and middle-income countries and an estimated 10 million new cases diagnosed annually [1]. In 2019, the worldwide economic cost of dementia was estimated at US $1.3 trillion [1]. Dementia is one of the most challenging health problems in later life that impacts individuals affected, as well as their families, caregivers, and society [2]. It is recognized as a public health priority, highlighting the need for a comprehensive public health approach and sustained advocacy to drive action at international and national levels [3].

Most people living with dementia live in the community, with care provided by informal caregivers, particularly family members [4]. They play a central role in supporting people living with dementia in the community, providing most day-to-day care that enables them to remain at home. According to the World Health Organization, approximately half of the global costs of dementia are attributable to informal care provided by family members and close friends [1]. Family caregivers are the primary source of practical, emotional, social, and economic support for people with dementia living in the community, helping to maintain their quality of life [5]. However, they encounter several challenges throughout their caregiving journey. They often feel unprepared and lack the knowledge and skills needed to provide daily dementia care, and most family caregivers receive little or no formal training [6-8]. Therefore, they require additional support, particularly in accessing reliable information about the condition, receiving training on how to cope with symptoms and behavioral problems, and identifying appropriate support services and systems to assist them in their caregiving role [2,7,9]. As people living with dementia exhibit a broad and heterogeneous range of symptoms and patterns of progression, which can vary greatly from one individual to another [10], each caregiver faces a different care journey. Accordingly, caregivers may need personalized guidance and resources to effectively manage their caregiving responsibilities.

Digital health technologies have been increasingly used to support family caregivers [11]. Conversational artificial intelligence (AI), particularly in the form of chatbot applications, is increasingly being explored in health care as a promising approach to support communication, education, and self-management [12,13]. These are software programs designed to simulate human conversation through text or voice interfaces by leveraging natural language processing and machine learning techniques [14]. The integration of chatbot applications into health education and support has become an increasingly prominent trend. Literature indicates that these applications enable the delivery of timely, personalized education and support [15], providing humanlike, personalized interactions to enhance health care accessibility and efficiency [16,17]. The effectiveness of chatbots has been demonstrated in various health care contexts, including the self-management of chronic diseases [18], patient education [19,20], changing behaviors [21], and medical [22] and nursing education [23,24]. Therefore, a chatbot can be considered an effective mode for providing education and support in health care.

AI solutions are trending in dementia care, and a recent systematic review has examined AI solutions for caregivers of people with dementia, giving special attention to their documented needs [25]. The main interfaces identified in this review were software applications, wearable devices, smart devices at home, and smart homes, while the main identified needs included supervision or support, emotional health, physical or nursing care, information about dementia and dementia care, household work, juggling responsibilities, relationships with the care recipient and with family, future planning, and information about professional support and formal services. Moreover, the literature reports that several chatbot applications have been developed to address the needs of caregivers of people with dementia, for example, “CareHeroes” in the United States [26], “Ana,” a chatbot in Peru [27], the “PDC30” chatbot in HongKong [28], “eDem CONNECT” in Germany [29], and “RecuerdameBot” in Spain [30]. A recent systematic review exploring commercially available chatbots for people with dementia and their caregivers found that these applications are increasingly used to provide support. However, their development and implementation remain at an early stage, with limited user testing and insufficient evidence regarding their effectiveness in providing education or support [31]. The literature further highlights the need to consider the dynamic nature of informal caregivers’ needs and to develop more caregiver-centered AI solutions [25], as well as more evidence-based chatbots with end user evaluation to assess their potential to adequately educate and support these populations [32]. Therefore, given the rapid expansion of chatbot technologies in health and social care, synthesis of evidence is essential to examine their use as educational and supportive tools for family caregivers of people with dementia. Examining current evidence on chatbot design, development, and effectiveness can guide the planning and implementation of future interventions. This systematic integrative review aims to synthesize evidence on chatbot applications as an educational and supportive tool for family caregivers of people with dementia.

### Review Questions

In this review, the population, phenomena of interest, and context [33] criteria will be used. The population is family caregivers of people with dementia. The phenomenon of interest is the use of chatbots as an educational and supportive digital intervention. The context of this study includes caregiving settings, including community, home care, and outpatient settings. Review questions were as follows: (1) What are the needs addressed in chatbot applications for family caregivers of people with dementia? (2) What is the development process followed, and what features and functions are included in chatbot applications for family caregivers of people with dementia? (3) What is the effectiveness or contribution of chatbot applications as an educational and supportive resource for family caregivers of people with dementia? (4) What are the barriers and challenges in designing and developing chatbot applications for caregivers of people with dementia? and (5) What are the ethical and safety concerns reported in the use of chatbots for caregivers of people with dementia?

## Methods

### Study Design

In this review, the integrative review framework proposed by Whittemore and Knafl [34] will be used. We selected this approach as it will enable researchers to include both qualitative and quantitative studies to identify, appraise, and synthesize the existing evidence on the use of chatbot applications as an educational and supportive resource for family caregivers of people with dementia. This approach enables a more comprehensive understanding by systematically integrating and interpreting empirical evidence [34] compared with a scoping review, which primarily aims to map the extent, nature, and characteristics of the available literature without critically synthesizing findings [35]. Furthermore, the integrative review approach facilitates the comparison of findings across studies; identification of patterns and relationships; and development of broader conclusions that can inform practice, policy, and future research.

The PRISMA-P (Preferred Reporting Items for Systematic Reviews and Meta-Analysis Protocols) 2015 checklist [36] guided the development of this protocol. This review was registered with PROSPERO (CRD420261288076).

### Literature Search

A total of 7 databases will be searched: MEDLINE via EBSCOhost, Embase, CINAHL via EBSCOhost, PsycInfo via EBSCOhost, Web of Science, Cochrane Library, and Scopus. A comprehensive search strategy will be developed using free-text keywords and Medical Subject Headings (MeSH) terms, combining three main concepts: (1) dementia, (2), family caregivers, and (3) chatbot technologies. Boolean operators (AND/OR) will be used to combine search terms. The following keywords will be used: (dementia OR Alzheimer*) AND (carer* OR caregiver* OR famil* OR spous* OR child* OR relati* OR friend* OR neighbour* or neighbor* OR supporter* OR helper*) AND (chatbot* OR “chat bot*” OR “chat-bot*” OR “conversational agent*” OR “virtual assistant*” OR “virtual agent”* OR “relational agent*” OR “generative artificial intelligence” OR “conversational artificial intelligence” OR “conversational AI” OR “conversational bot*” OR “conversational system*” OR “conversational interface*” OR chatterbot* OR “large language model*” OR LLM* OR “digital determinant* of health” or “digital health equity”). MeSH terms including “dementia,” “Alzheimer disease,” “caregivers,” “spouses,” “family,” and “artificial intelligence” will be used. A full search strategy for the MEDLINE database is presented in Multimedia Appendix 1. The search strategy will be adapted for other databases. Moreover, gray literature will be searched for additional empirical studies using Google Scholar and ProQuest Dissertations and Theses Global.

This review has been planned as a static systematic integrative review. We will search the literature up to 2026, and no updates are planned at this stage. After completing data extraction, synthesis, and quality assessment of all eligible studies identified up to this date, the review will be finalized. The findings of this study may inform future research projects, such as the development of chatbots for caregivers of people with dementia. If further updates are required for subsequent research projects, we will follow the same systematic search, screening, data extraction, quality appraisal, and synthesis procedures as outlined in this protocol.

### Inclusion and Exclusion Criteria

In this systematic integrative review, we will include peer-reviewed, full-text articles based on primary studies (quantitative, qualitative, and mixed methods) published in English from inception to 2026 on chatbot applications for caregivers of people with dementia.

In this review, we will include conversational software applications, such as rule-based, AI-driven, or hybrid chatbots designed to simulate human interaction through natural language dialogue, enabling users to communicate with digital systems via text or voice interfaces. Any articles that include family caregivers together with other populations (eg, people with dementia) will also be included. Articles will be included if they are related to the design, development, and evaluation of a chatbot application for family caregivers of people with dementia. Studies that primarily focus on education, such as providing knowledge and skills, or on support, including caregiving assistance, emotional support, and guidance, will be included. Additionally, studies examining usability, feasibility, acceptability, utility, adoption, and effectiveness (eg, reducing stress and burden or improving caregiving knowledge) will also be considered. In addition, we will include articles that identify potential barriers and challenges, as well as ethical and safety concerns, related to the design, development, and implementation of chatbot applications for family caregivers of people with dementia. Studies related to nonconversational digital tools, automated messaging systems, and human-only communication systems will be excluded. Moreover, articles not published in English or not available in full text, as well as editorials, study protocols, review articles, opinion and perspective papers, and technical notes, will be excluded. Moreover, articles focusing on chatbot applications only for people with dementia will be excluded. We will review the reference lists of included reviews to minimize the risk of missing relevant studies.

### Search Outcomes

The primary outcome of this study is the effectiveness of the chatbot application as an educational and supportive resource for family caregivers of people with dementia. The intervention will be assessed in terms of usability, feasibility, acceptability, utility, adoption, and effectiveness in relation to education and support. Other outcomes will include identifying needs addressed in chatbot applications, the development process followed, the features and functions of chatbot applications, the barriers and challenges, and ethical and safety concerns in developing chatbot applications for family caregivers of people with dementia.

### Data Screening

An information specialist will conduct the database search, and all sources will be included in Covidence. Covidence software will be used to remove duplicates; screen articles by title, abstract, and full text; and perform data extraction. A minimum of 2 reviewers will be involved in screening titles, abstracts, and full texts. The number of included and excluded studies at each stage of the study will be reported using a PRISMA (Preferred Reporting Items for Systematic Reviews and Meta-Analyses) flow diagram [37].

### Data Extraction

A data extraction form (Excel sheet) will be developed and piloted on at least 3 full-text papers by at least 2 independent reviewers to ensure consistency and completeness of the form. It will be refined based on the piloting process. Information from all included studies will be extracted using Covidence software, including the details of authors, publication year, country, title, study aims, theoretical or conceptual frameworks, design, methods, and key findings.

### Risk of Bias Assessment

Two independent reviewers will assess the papers meeting the inclusion criteria for methodological quality and data relevance using a method suggested for integrative reviews by Whittemore and Knafl [34]. The Mixed Methods Appraisal Tool (version 2018) [38] will be used to assess the methodological quality. This tool will help evaluate the risk of bias in different study types, including qualitative studies, quantitative randomized trials, quantitative nonrandomized studies, quantitative descriptive studies, and mixed methods studies. Two reviewers will independently appraise the quality, and any discrepancies will be resolved through discussion or consultation with a third reviewer. To assess data relevance, a 2-point scale (high or low) will be used [34], in which high means that the study’s findings are highly relevant to the review question and low means the study’s findings are only marginally relevant or not directly useful.

### Data Synthesis

In this systematic integrative review, a narrative synthesis will be conducted to integrate the identified evidence from both qualitative and quantitative studies. The analysis will follow a process for integrative literature reviews, including data reduction, data display, data comparison, and conclusion drawing and verification, as outlined by Whittemore and Knafl [34]. Key characteristics of the findings of included studies will be organized into evidence tables to facilitate data reduction and comparison. Quantitative and qualitative findings will first be examined separately to summarize study characteristics, interventions, outcomes, and key themes. Then, these findings across different study types will be compared and integrated through narrative synthesis. When synthesizing ethical and safety concerns, we will use the framework proposed by the World Health Organization, “Ethics and Governance of Artificial Intelligence for Health” [39]. We will follow the core principles outlined in this guidance, including protecting human autonomy, promoting human well-being and safety, ensuring transparency, fostering responsibility and accountability, ensuring inclusion and equity, and supporting responsive and sustainable AI.

Text and tables will be used to present results. All eligible studies will be included in the systematic integrative review based on their relevance to the review question [34], regardless of their risk of bias as determined by the quality assessment. Therefore, studies whose findings are highly relevant to the review question based on information value (ie, those achieving a score of 2 on the relevant criteria) [34] will be included. If a sufficient number of high-quality randomized controlled trials are found, a meta-analysis will be conducted as a subgroup analysis. Only trials with comparable interventions, populations, and outcome measures will be pooled for meta-analysis. Confidence in the cumulative evidence will be assessed using the Grading of Recommendations, Assessment, Development, and Evaluation approach [32].

### Dissemination of Findings

The results of this review will be presented at national and international conferences or forums and published in a peer-reviewed journal.

### Ethical Considerations

Ethics approval is not required for this research, as we will use published literature.

## Results

**Figure 1:**
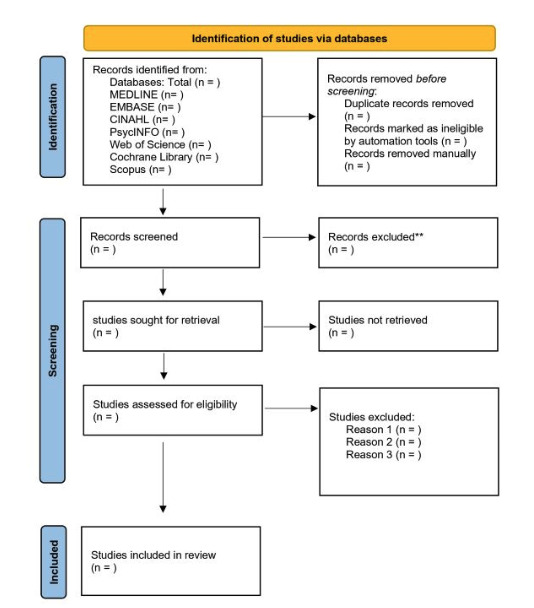
PRISMA (Preferred Reporting Items for Systematic Reviews and Meta-Analyses) flow diagram.

The literature search is scheduled to commence in May 2026. Title and abstract screening and full-text review are expected to be completed between June 2026 and September 2026 using the PRISMA flow approach [37], as shown in Figure 1. Data extraction and synthesis will be conducted from September to November 2026. The findings are expected to be submitted for publication as a systematic integrative literature review in January 2027.

## Discussion

### Anticipated Findings

The rapid expansion of digital health technologies has created new opportunities in dementia care, particularly for supporting family caregivers of people living with dementia. Chatbot applications, as conversational and interactive digital interventions, have the potential to provide timely, personalized, and scalable education and support tailored to caregivers’ needs [15-17]. However, despite growing interest in chatbot-based interventions, particularly in dementia care focusing on family caregivers, the current evidence is limited regarding needs addressed, design approaches and frameworks followed, functionality, evaluation methods, and reported outcomes. This systematic integrative review will aim to address this gap by synthesizing existing evidence on the needs of caregivers, development processes, effectiveness and challenges, barriers, and ethical and safety concerns associated with chatbot applications for family caregivers of people with dementia.

This review will integrate qualitative, quantitative, and mixed methods studies, thereby providing a comprehensive understanding of how chatbot interventions are conceptualized, developed, and evaluated in dementia caregiving contexts. In line with the integrative review methodology [34], the inclusion of diverse study designs will allow for an in-depth exploration of caregivers’ needs, framework followed, development process, barriers and challenges, and ethical and safety concerns in developing and designing chatbot applications and their contribution. Identifying barriers, challenges, and ethical and safety concerns encountered in the design, development, and implementation of chatbot applications for family caregivers will help researchers, developers, and policymakers anticipate and address factors that could limit real-world design, development, implementation, and sustainability of chatbot applications for family caregivers of people with dementia.

### Limitations

We will exclude literature published in languages other than English and will search only 7 databases, which may limit some literature and add limitations to this study. Despite these limitations, this integrative review will provide implications for the development and implementation of chatbots for family caregivers of people with dementia.

### Implications

The findings of this systematic integrative review are expected to have important implications for research, practice, and policy. For researchers, this review will identify methodological strengths and limitations in the existing literature; highlight key priorities; and identify barriers and challenges, as well as ethical and safety concerns, that need to be addressed in future studies on the development and design of chatbot applications for family caregivers of people with dementia. For service providers, the findings may help identify caregivers’ needs and inform the adaptation and co-design of chatbot interventions that better align with those needs. At a policy level, the findings may support informed decision-making regarding the design and implementation of AI-based interventions, particularly in relation to regulation, ethical governance, and quality assurance for chatbot-based digital health interventions targeting vulnerable populations, such as family caregivers of people with dementia.

### Conclusions

Systematic reviews are widely regarded as one of the most robust sources of research evidence and have played a key role in informing health care decision-making [40]. To the authors’ knowledge, this is the first study to systematically analyze and synthesize evidence on chatbot applications for family caregivers of people with dementia, examining the needs addressed, development processes, contributions, and effectiveness of these applications in dementia care, as well as the challenges and barriers, and ethical and safety concerns, to their development and implementation. This review will provide deeper insights into the design and development of chatbot interventions for caregivers of people with dementia.

## Data Availability

No datasets were generated or analyzed during this study; therefore, data sharing is not applicable.

## Appendix

Multimedia Appendix 1 Literature search strategy: MEDLINE via EBSCOhost.

## Abbreviations

AI: artificial intelligence
MeSH: Medical Subject Headings
PRISMA: Preferred Reporting Items for Systematic Reviews and Meta-Analyses
PRISMA-P: Preferred Reporting Items for Systematic Reviews and Meta-Analysis Protocols
