# Supplementary material for: Evolution of Chatbots as an Educational and Supportive Digital Intervention for Family Caregivers of People With Dementia: Protocol for a Systematic Integrative Review
Source: JMIR Res Protoc. 2026 Jun 9;15:e91741. doi: 10.2196/91741 (PMC13291734; doi:10.2196/91741)
Supplement: Multimedia Appendix 1 [file resprot_v15i1e91741_app1.docx]

**Table 1**: Literature search strategy: MEDLINE via EBSCOhost

| # | Query | Results |
| --- | --- | --- |
| S14 | S3 AND S6 AND S13 | xxx |
| S13 | S7 OR S8 OR S9 OR S10 OR S11 OR S12 | xxx |
| S12 | "virtual assistant*" OR "virtual agent*" OR "relational agent*" OR "generative artificial intelligence" | xxx |
| S11 | "conversational agent*" OR "conversational artificial intelligence" OR "conversational AI" OR "conversational bot*" OR "conversational system*" OR "conversational interface*" | xxx |
| S10 | "large language model*" OR LLM* | xxx |
| S9 | chatbot* OR "chat bot*" OR "chat-bot*" OR chatterbot* | xxx |
| S8 | ("digital determinant*" N2 health") OR" digital health equity" | xxx |
| S7 | MH "Large Language Models" OR MH "Generative Artificial Intelligence" | xxx |
| S6 | S4 OR S5 | xxx |
| S5 | dement* OR alzheimer* | xxx |
| S4 | MH "Alzheimer Disease" OR MH "Dementia" | xxx |
| S3 | S1 OR S2 | xxx |
| S2 | carer* OR caregiver* OR famil* OR spous* OR child* OR relati* OR friend* OR neighbour* OR neighbor* OR supporter* OR helper* |  |
| S1 | MH "Family Support" OR MH "Caregivers" OR MH "Friends" | xxx |
